# Sleep Changes in Adolescents Following Procedural Task Training

**DOI:** 10.3389/fpsyg.2016.01555

**Published:** 2016-10-06

**Authors:** Rebecca S. Nader, Anthony L. Murkar, Carlyle T. Smith

**Affiliations:** ^1^Department of Psychology, Trent UniversityPeterborough, ON, Canada; ^2^Department of Psychology, University of OttawaOttawa, ON, Canada

**Keywords:** stage 2, rem, SWS, spindles, adolescents, learning

## Abstract

Recent research has suggested that some of the inter-individual variation in sleep spindle activity is due to innate learning ability. Sleep spindles have also been observed to vary following learning in both young and older adults. We examined the effect of procedural task acquisition on sleep stages and on sleep spindles in an adolescent sample. Participants were 32 adolescents (17 females) between the ages of 12 and 19 years. Spindle activity was examined in three different frequency ranges: 11.00–13.50 Hz (slow), 13.51–16.00 Hz (fast), and 16.01–18.50 Hz (superfast). No changes in spindle density were observed after successful learning of the pursuit rotor task. This result was in contrast to a number of studies reporting spindle density increases following successful learning. In the present study, participants who successfully learned the task showed no changes in their sleep stage proportions, but participants who were not successful showed a decrease in the proportion of stage 2 and increases in both SWS and REM sleep. We suggest that these changes in the sleep stages are consistent with the two stage model of sleep and memory proposed by Smith et al. (2004a).

## Introduction

Sleep spindles are a hallmark of stage 2 sleep, often used as the defining characteristic of stage 2 onset. They are commonly considered to have a frequency range of 11–16 Hz; this range is often further divided into two types of sleep spindles, with slow spindles having a frequency range of approximately 11–13.5 Hz and fast spindles having a frequency range of 13.5–16 Hz (Zeitlhofer et al., 1997; DeGennaro and Ferrara, 2003; Fogel and Smith, 2011; Nader and Smith, 2015). We have previously identified what we believe to be a third spindle type in the frequency range of 16–18.5 Hz (Nader and Smith, 2015).

Along with the traditional slow and fast spindles, these ‘superfast’ spindles (16–18.5 Hz) were observed to appear in all sleep stages in a sample of healthy adolescent males and females (Nader and Smith, 2015). The superfast spindle was observed in all of our adolescent subjects, albeit with a lower occurrence than either the slow or fast spindles. All three types of spindles were observed in all sleep stages, despite the common acceptance that sleep spindles are primarily a stage 2 phenomenon (Rechtschaffen and Kales, 1968; Steriade and McCarley, 2005). Using automated spindle counters allowed us to filter out electroencephalography (EEG) frequencies that were not of interest to us, and to focus on specific frequency ranges (Ray et al., 2009). This has resulted in better detection of the various types of spindles, as well as increased detection in sleep stages other than stage 2 including REM sleep (Gaillard and Blois, 1981; Zeitlhofer et al., 1997; Nader and Smith, 2015).

Sleep spindles, while being very consistent within an individual, have large inter-individual differences (Gaillard and Blois, 1981; DeGennaro et al., 2005; Fogel and Smith, 2011). Recent research has suggested that the sleep spindle is linked with cognitive ability both in children (Hoedlmoser et al., 2014) and adults (Nader and Smith, 2001; Bódizs et al., 2005; Schabus et al., 2006). Higher levels of spindle activity have been observed to be positively related to a number of tasks that measure various aspects of cognitive ability. Schabus et al. (2006) observed more slow and fast spindle activity (amplitude × duration) in individuals who scored highly on the Raven’s Progressive Matrices (a measure of cognitive ability) and in individuals who scored highly on the Wechsler Memory Scale (a measure of memory performance). Bódizs et al. (2005) observed a similar effect using the Raven’s Progressive Matrices, but they found that only the fast spindles were positively correlated with performance; the density of the fast spindles were found to explain nearly 70% of the variance in performance on the cognitive test. Other studies have provided evidence supporting the hypothesis that baseline spindle activity is positively linked to cognitive abilities or learning potential (Nader and Smith, 2001, 2003, 2015; Fogel et al., 2007b; Hoedlmoser et al., 2014).

Many researchers have also explored whether spindle activity is affected by learning. To this end, researchers have employed a number of tasks to try and induce learning-dependent changes in spindle activity. Fogel and Smith (2006) found that young adults exhibited a 42% increase in the number of sleep spindles, and a 24% increase in spindle density after learning four different motor tasks. Peters et al. (2008) observed a significant increase in spindle density in stage 2 sleep after participants learned the pursuit rotor task – this increase was present in younger adults (17–24 years), but not in the older adults (62–79 years). These researchers suggest, however, that it was performance that was important, not age *per se*; the participants who learned, exhibited an increase in spindle density, but those who did not learn did not show an increase in spindle activity (Peters et al., 2008). It may also be that increases in spindle activity after learning may be dependent on intelligence, with the effect being seen primarily in those individuals with higher IQs (Fogel et al., 2007a; Schabus et al., 2008).

Other studies have suggested that children may be different than young adults with respect to the sleep spindle changes observed following learning. Hoedlmoser et al. (2014) used a declarative task in a group of pre-pubertal children rather than a procedural task, but they did not observe any relationship between memory consolidation and increased spindle activity. Similar to adults, general cognitive abilities did seem to be related to spindle activity, but learning itself did not induce any changes in spindle activity. This may have a number of implications: for example, it may be that in terms of sleep states, the declarative task does not depend on spindle activity for consolidation, the task may have been too difficult for the children (as suggested by the authors) or it may be that children respond differently than adults to learning.

Smith et al. (2004a) have previously proposed a two stage model of motor learning. In this model, the sleep stages involved in memory acquisition depend upon the level of task mastery. In this model, when a task is novel to an individual, successful post-acquisition changes in REM sleep are observed; however, when a task is familiar to the individual and he/she is simply refining a skill, then successful post- acquisition changes in stage 2 sleep are more likely. Thus, it is possible that any sleep changes observed in our adolescent sample may be dependent on their familiarity with the task/skill set.

The present study was focused on investigating the link between spindle activity and motor learning in adolescents. We recorded the sleep of adolescents both before and after acquisition of a simple motor task (the pursuit rotor). We were interested in the link between baseline sleep measures and learning; we anticipated that learning would be positively related to an increase in spindle activity from baseline to post-learning (PL) sleep. Based on the findings of Ujma et al. (2014), we were also interested in whether there were gender differences in the relationships between sleep and learning.

## Materials and Methods

### Participants

The participants were 32 adolescents (17 females) between the ages of 12 and 19 years (*M* = 15.36 years) recruited from the Peterborough community. Participants were all considered to be healthy and medication free, as assessed by their parents, with no indication of sleep disorders. As well, all participants attended regular school class programs. All subjects were assessed for pubertal development, using the Tanner Scale, in order to statistically control for hormonal effects on spindle activity if necessary. This study was approved by the Trent University Research Ethics Board.

### Measures

Electroencephalography in-home recordings were made using Suzanne^TM^ (Tyco-Healthcare Group LP, Mansfield, MA, USA) portable polysomnographic systems. The sampling rate was 120 Hz and data were stored on PC flash memory cards, and then downloaded off-line onto a PC computer for further analysis. We recorded EEG, electrooculogram (EOG) (horizontal eye movements only), and EMG using silver-plated electrodes. The EEG (C3, C4, FZ, and PZ) and the EOG (right and left eyes) were monopolar recordings and referenced to contralateral electrodes at A1 and A2. The EMG channel was bipolar. For the EEG and EOG channels, the low-and high-pass software filters were set at 0.03 and 30 Hz. For the EMG channel, only frequencies above 10 Hz were recorded.

Three consecutive nights of in-home sleep recording were carried out. The data from recording night 1 were considered to reflect acclimatization to the apparatus and were discarded. Night 2 recordings were used as Baseline sleep data and night 3 recordings were used as PL sleep data. Participants were asked to adhere to their normal bedtime routines as much as possible, including keeping their usual bedtime and wake time.

Sleep stages were generally scored according to standard criteria (Rechtschaffen and Kales, 1968). However, we sometimes deviated slightly from traditional protocol when scoring the REM sleep stage. The appearance of spindles during REM sleep in the raw EEG was rare, and they only became more visible in the filtered channel. However, according to standard criteria, the observation of a spindle would normally signal an ending to the REM period and the beginning of a period of stage 2 with the appearance of other stage 2 indicators. It would also be expected that there would be some increased activity in the EMG channel. If there was absolutely no change in the EMG, no other sign of a stage 2 intrusion (such as a K- complex) and further REM bursts, the epoch was counted as REM sleep despite the appearance of a spindle. Sleep spindles were counted using the automated spindle counter PRANA^®^ (PhiTools, Strasbourg, France). For each spindle type, an expert technologist identified and recorded the peak amplitudes of 15 spindles in each of the first and second halves of the night for stage 2 (30 spindles in total for each spindle type). Values were then used to calculate the mean and standard deviation of peak amplitude for each subject. The minimal amplitude criterion for the automated spindle counter was determined by subtracting 1.96 SD units from each mean. This procedure was repeated for each subject. Spindle activity was examined in each of the 11.00–13.50, 13.51–16.00, and 16.01–18.50 Hz range. Included in the study were spindle-like waves in the 16.01–18.50 Hz range. These waves share many characteristics of conventional spindle appearance and activity. We have previously described the properties of these waves and consider them to be a special spindle subset called ‘superfast’ spindles (Nader and Smith, 2015). This EEG activity appears to varying degrees in all individuals.

Intelligence was assessed using the Wechsler Intelligence Scale for Children-Fourth Edition (WISC-IV) Canadian Edition. Tests were administered individually by a registered psychometrist. Five participants were assessed by the same psychometrist using the Wechsler Adult Intelligence Scale—Third Edition (WAIS-III) as they were above the age for the WISC-IV.

**FIGURE 1:**
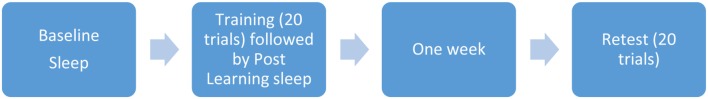
**Visual description of study design**.

Prior to the third night of sleep, participants were administered the Pursuit Rotor task approximately two hours prior to normal bedtime, using an online program and a laptop computer (Fogel et al., 2007b). The pursuit rotor task requires participants to use the mouse to keep the cursor on a light as it travels around a path on the computer screen. Participants completed twenty 30-s trials prior to sleep on the third night. All subjects were able to perform the pursuit rotor task with no physical difficulty. The sleep recorded the night after acquisition of the task, was considered a post-training night which allowed us to determine whether there were any sleep-related changes due to acquisition of the learning task. They were retested exactly one week later, with another 20 trials (see **Figure 1**).

## Results

### Learning Task

The measure used to assess degree of learning was the number of seconds that the subject was able to keep the cursor on a lighted dot as it moved around the path.

When all of our participants were used, a *t*-test revealed that there was a significant improvement in scores from the last 12 training trials (*M* = 4.76 s, *SD* = 2.19) to the first 12 re-test trials (*M* = 5.79 s, *SD* = 2.58), *t*(32) = -3.66, *p* = 0.0009, *d* = -0.66. Results indicate that the participants did successfully learn the pursuit rotor task.

A closer examination of the learning scores suggested that some individuals did not learn the task. Participants were then split into good performers vs. poor performers, using a median split on the assessed degree of improvement. A subsequent *t*-test showed that there was a significant difference in performance between the two groups, *t*(31) = -7.37, *p* < 0.00001, *d* = -2.60, with the good performers demonstrating significantly more improvement (*M* = 2.28 s, *SD* = 1.11) than the poor performers (*M* = -0.28 s, *SD* = 0.86).

### Pubertal Development

Participants were assessed for the level of pubertal development using the Tanner Stages. To determine whether pubertal development was playing a role in performance on the pursuit rotor task, the score on the Tanner scale was correlated with our performance measure. There was no significant relationship between pubertal development and PR performance, *r*(29) = -0.20, *p* = 0.29. We also assessed whether the pubertal development was related to baseline sleep. There were no significant correlations between scores on the Tanner scale and percentage of stage 2, *r*(29) = 0.29, *p* = 0.11, percentage of SWS, *r*(29) = -0.18, *p* = 0.35, or percentage of REM, *r*(29) = -0.16, *p* = 0.39. Participants were separated by gender to ensure that there were no relationships between pubertal development and sleep and there were none found for females or males between Tanner scale and percentage of stage 2 [*r*(15) = 0.20, *p* > 0.05; *r*(12) = 0.38, *p* > 0.05, respectively], percentage of SWS [*r*(15) = -0.11, *p* > 0.05; *r*(12) = -0.26, *p* > 0.05, respectively] or percentage of REM [*r*(15) = -0.06, *p* > 0.05; *r*(12) = -0.26, *p* > 0.05, respectively].

### Sleep Measures

The proportion of the night spent in each sleep stage was assessed prior to learning and after task acquisition (**Table 1**). SWS is composed of stages 3 and 4 combined as defined by Rechtschaffen and Kales (1968).

**Table 1:** Mean and standard deviation of the percentage of time spent in sleep stages before and after learning (all participants).

	Stage 2%	SWS%	REM%
Baseline night	50.47 (6.87)	25.35 (6.56)	23.03 (5.48)
Post-learning night	47.67 (5.86)	26.13 (6.01)	25.02 (3.65)

### Learning and Sleep Measures

To test whether the level of learning was related to sleep, we examined the correlations between learning and sleep stage proportions. To begin, we examined the correlations between baseline sleep and how well-participants learned the pursuit rotor task. There were no correlations between stage 2% [*r*(31) = -0.12, *p* = 0.50], SWS% [*r*(31) = 0.13, *p* = 0.47] or REM% [*r*(31) = -0.01, *p* = 0.94], and performance on the learning task. Baseline sleep did not predict future learning.

The correlations were repeated separately for females and males. While neither gender showed significant relationships between percentage of the sleep stages and performance on the learning task, they did seem to show different patterns of relationships. Neither females nor males showed a significant correlation between baseline percentage of stage 2 and performance on the pursuit rotor [*r*(15) = 0.24, *p* > 0.05; *r*(13) = -0.36, *p* > 0.05, respectively], or percentage of SWS and pursuit rotor performance [*r*(15) = 0.08, *p* > 0.05; *r*(13) = 0.15, *p* > 0.05, respectively], or percentage of REM and pursuit rotor performance [*r*(15) = -0.33, *p* > 0.05; *r*(13) = 0.29, *p* > 0.05, respectively]. While none of these are significant, the differences between the males and females in the relationships between REM and stage 2 with pursuit rotor performance should be further investigated with more participants.

**FIGURE 2:**
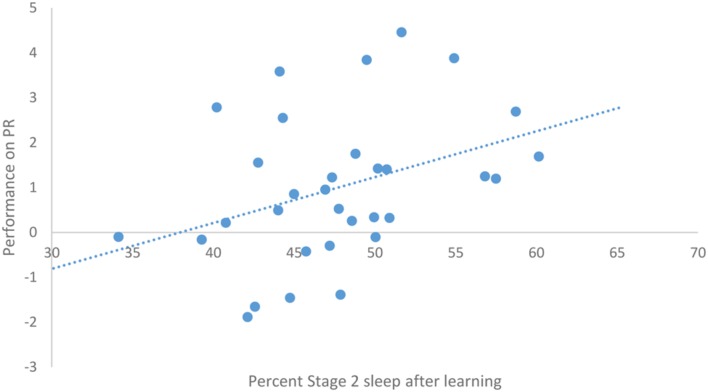
**Correlation between proportion of stage 2 sleep on PL night and performance on pursuit rotor (measured as improvement between training and testing)**.

**FIGURE 3:**
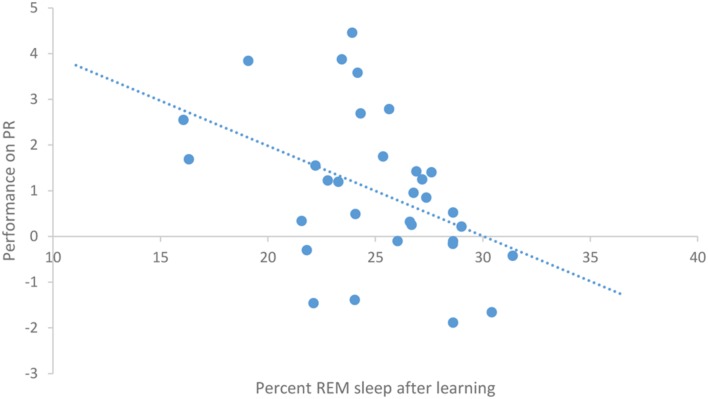
**Correlation between proportion of REM sleep on PL night and performance on pursuit rotor (measured as improvement between training and testing)**.

After task acquisition on night 3 (PL night), we observed a significant positive relationship between the proportion of stage 2 sleep and how well-participants learned the pursuit rotor, *r*(30) = 0.37, *p* = 0.039 (see **Figure 2**). There was also a significant negative correlation between the percentage of REM sleep and how well-participants learned, *r*(30) = -0.44, *p* = 0.011 (see **Figure 3**). There was no significant correlation between proportion of SWS on PL Night and how well-participants learned, *r*(31) = -0.08, *p* = 0.68. These relationships suggest while there was no relationship with baseline sleep and future learning, better performance on the pursuit rotor was associated with higher levels of stage 2 sleep and lower levels of REM sleep after learning.

Again, the correlations were repeated for the females and males separately, to ensure that there were no gender differences. Both genders showed a similar pattern of relationships; both females and males showed a positive relationship (albeit not significant) between proportion of stage 2 sleep after learning and how well-participants learned the pursuit rotor task [*r*(15) = -0.33, *p* > 0.05; *r*(13) = 0.41, *p* > 0.05, respectively]. Both females and males showed no relationship between proportion of SWS on PL Night and how well they learned [*r*(15) = -0.06, *p* > 0.05; *r*(13) = -0.08, *p* > 0.05, respectively]. Females showed a significant negative relationship between the proportion of REM sleep on the PL Night [*r*(15) = -0.55, *p* = 0.02], but the relationship for males was not significant [*r*(13) = -0.41, *p* > 0.05].

Examination of the sleep stage proportions (minutes of particular sleep stage/total sleep) for all participants, showed no significant differences between Baseline and PL night in either percentage of SWS (25.35 and 26.13%, respectively), *t*(31) = -1.03, *p* = 0.31, *d* = -0.18, or percentage of REM sleep (23.03 and 25.02%, respectively), *t*(31) = -1.97, *p* = 0.06, *d* = -0.36. However, the change in percentage of REM sleep showed a strong trend toward an increase in REM sleep after learning. When examined separately, neither females nor males showed a significant change in percentage of SWS from Baseline to PL night [*t*(16) = -0.79, *p* > 0.05; *t*(14) = -0.65, *p* > 0.05, respectively]. The increase in REM from Baseline to PL night was not significant in either females or males [*t*(16) = -1.32, *p* > 0.05; *t*(14) = -1.43, *p* > 0.05, respectively]. There was a significant decrease in the proportion of stage 2 from Baseline (50.47%) to PL night (47.67%), *t*(31) = 2.22, *p* = 0.03, *d* = 0.40. Females and males both showed similar decreases in the proportion of stage 2 from Baseline to PL night, although neither reached significance [*t*(16) = 1.47, *p* > 0.05; *t*(14) = 1.61, *p* > 0.05, respectively].

**FIGURE 4:**
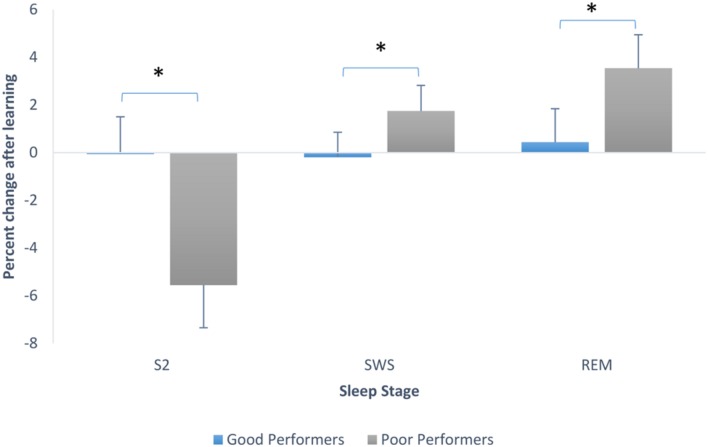
**Sleep stage percent changes from Baseline to PL night (after learning), separated by good performers and poor performers (bars indicate SE)**. SWS refers to combined S3 + S4.^∗^*p* < 0.001.

To explore this further we looked at sleep stage proportions based on degree of learning of the pursuit rotor task, using the median split on performance scores. Good performers had a mean of 2.28 (*SD* = 1.11) and poor performers had a mean of -0.28 (*SD* = 0.86). We ran an ANOVA testing whether there was an interaction between sleep stage percent changes from Baseline to PL night and whether or not participants learned. There was a significant main effect of stage, *F*(2,60) = 4.24, *p* = 0.019, and a significant interaction between stage and whether or not learning occurred, *F*(2,60) = 3.71, *p* = 0.03. *Post hoc* Tukey tests revealed that participants who did not perform well on the pursuit rotor task showed a greater decline in the proportion of stage 2 sleep than those who showed better performance on the task (these last mentioned participants actually showed no change in proportion of stage 2). *Post hoc* tests also showed that poor performing participants also showed a larger increase in the proportion of SWS than participants who learned (good performers again, remained very consistent in the proportion of SWS from Baseline to PL night. Tukey tests also showed that participants who did not perform as well-increased the proportion of REM sleep significantly in comparison to those participants who showed better mastery on the PR. Good performers again, remained steady in the proportion of this sleep stage. **Figure 4** demonstrates the changes in sleep stage proportions.

### IQ and Sleep Measures

To begin, we correlated full scale IQ (FSIQ; *M* = 98.55, *SD* = 8.56) with the performance on the pursuit rotor task (measured as the difference between training and testing). FSIQ was not significantly related to performance, *r*(31) = 0.20, *p* = 0.28.

Using a median split of FSIQ, we examined the changes in sleep stage proportions from Baseline to PL night. Lower IQ individuals had a mean IQ of 91.81 (*SD* = 4.98) and the higher IQ individuals had a mean of 104.88 (*SD* = 5.94). The results were very similar to those observed using performance scores. An ANOVA was run testing whether there was an interaction between sleep stage percent changes from Baseline to PL night and IQ. There was a significant main effect of stage, *F*(2,60) = 4.20, *p* = 0.02, but no significant effect of FSIQ, *F*(1,30) = 0.23, *p* = 0.64 and no significant interaction between stage and FSIQ, *F*(2,60) = 1.26, *p* = 0.29. A *post hoc* Tukey test revealed that there was a significant difference between the change in the proportion of stage 2 and the change in the proportion of REM sleep. The proportion of stage 2 sleep declined by 2.8% and the proportion of REM sleep increased by 1.99%.

### Spindle Densities

To examine the hypothesis that learning would be related to spindle activity, we examined the correlations between spindle densities on PL night and learning but observed no significant relationships. Spindle density was chosen as the best way to assess spindle activity, because it allows us to easily compare across individuals, as it takes the time spent in stage 2 into account; it is the only measure which can truly assess an increase in the output of any spindle generator. Number of spindles alone may simply mean that there was an increase in stage 2 sleep. To confirm that there were no changes in spindle activity related to learning, we examined the spindle density differences from Baseline to PL night using three ANOVAs. The slow spindle density differences (11–13.5 Hz) were examined first in a 2 (poor performers vs. better performers) × 4 (electrode location) ANOVA, there was no main effect of learning, *F*(1,25) = 0.64, *p* = 0.43, and no main effect of electrode location, *F*(3,75) = 0.10, *p* = 0.96, and no interaction, *F*(3,75) = 2.57, *p* = 0.06. The fast spindle density differences (13.51–16 Hz) were also examined in a 2 (poor performers vs. better performers) × 4 (electrode location) ANOVA, similar to slow spindles, there was no main effect of learning, *F*(1,25) = 0.74, *p* = 0.40, and no main effect of electrode location, *F*(3,75) = 1.54, *p* = 0.21, and no interaction, *F*(3,75) = 0.60, *p* = 0.62. The superfast spindle differences (16.01–18.5 Hz) were also examined in a 2 (good performers vs. poor performers) × 4 (electrode location) ANOVA, similar to slow and fast spindles, there was no main effect of learning, *F*(1,25) = 0.15, *p* = 0.70, and no main effect of electrode location, *F*(3,75) = 0.47, *p* = 0.70, and no interaction, *F*(3,75) = 0.45, *p* = 0.72.

## Discussion

The pursuit rotor task is a well-used task for procedural learning (Smith and MacNeill, 1994; Smith et al., 2004b; Fogel and Smith, 2006; Peters et al., 2007). Researchers using the pursuit rotor have demonstrated good learning with the task (e.g., Peters et al., 2007), our participants did show significantly improved performance at re-test time, but they did not seem to show exceptionally high scores. Participants may not have demonstrated optimal performance due to the nature of the task. It was not a particularly engaging task for adolescents who were likely used to much more action-oriented video games with elaborate color graphics. It is suggested for future research that a more engaging procedural task be used with adolescent participants to ensure maximum attention and engagement with the task.

Our findings of no relationship between spindle density changes and learning, while not what we predicted, are similar to the findings of Peters et al. (2008) who found that spindle density changes were dependent upon learning after task acquisition. Our participants did learn, but the increase in performance was not very large and this may explain why we did not observe a significant relationship between spindle activity and performance on the pursuit rotor. In order to induce larger changes in spindle density a more intensive task may be required, or simply one that is more engaging for the adolescent population.

Our baseline measures of sleep suggest that the adolescents in our group had normal sleep stage proportions prior to learning the task (Coble et al., 1984). We ran correlations to examine the link between baseline sleep measures and learning potential (measured as performance on the pursuit rotor). Baseline sleep measures were unable to predict later performance on the pursuit rotor task, suggesting that ability to perform this simple procedural task cannot be inferred from an individual’s normal sleep patterns. These findings of no relationship between the baseline number of spindles and performance on the pursuit rotor, is in agreement with the findings of Peters et al. (2008) who also found that there was no correlation between baseline spindle density and motor performance. On the other hand, they are at variance with the results reported by Fogel et al. (2007b) and Fogel and Smith (2011). It is possible that with a more intensive learning paradigm, we may have seen some effects.

We did not observe any significant correlations between baseline sleep measures and motor performance when we separated our group by gender. However, males and females appeared to show opposite tendencies in the relationship between baseline REM and future motor performance and in the relationship between baseline stage 2 and future performance. Males showed a tendency toward a negative relationship between the proportion of baseline stage 2 and motor learning, whereas females showed a tendency toward a positive relationship between the variables. In contrast, males showed a tendency toward a positive relationship between the proportion of baseline REM sleep and motor performance, whereas females showed a tendency toward a negative relationship. These seemingly opposite tendencies may be due to a number of things. Ujma et al. (2014) suggest that there is a sexual dimorphism in the relationship between sleep spindle parameters and intelligence and our findings may similarly show that there are differences between the genders in the mechanisms responsible for learning. However, it would be inappropriate to speculate further on these non-significant results.

The positive correlation between percentage of stage 2 after learning and performance on the pursuit rotor, suggests that better learning is associated with higher levels of stage 2. However, these results need some qualification. We observed that participants who did not learn the task showed a decrease in the proportion of stage 2 sleep, whereas individuals who did learn the task simply maintained the percentage of stage 2 sleep that they had before learning the task. This suggests that while better learning is associated with higher levels of stage 2, it is actually because those who did not learn spent less time in that stage.

While the expected increases in spindle activity with learning did not occur in these adolescents, there was an interesting set of sleep changes that were clearly consistent with the two stage model of Smith et al. (2004a). In this model, participants exposed to a motor learning task with which they generally had had some previous experience show PL increases in stage 2 and density of stage 2 sleep spindles but no changes in REM sleep. These individuals appear to be refining a motor program that is already in place. However, participants that find the task to be new and novel show PL increases in REM sleep, but no changes in stage 2. These last individuals appear to need a new conceptual approach to learn the task, and this is reflected in an increase in REM sleep.

Examining all participants, we observed a decrease in the proportion of stage 2 sleep and a trend toward an increase in the proportion of REM sleep. By splitting the participants into those that performed better on the task and those who had poor performance in comparison, the changes in sleep become more obvious. While the participants in the present study that performed better on the pursuit rotor task showed no stage 2 or REM sleep changes, the poor performers did show these kinds of changes. As might be expected of participants that found the task extremely difficult and required a new cognitive approach, the non-learners showed an increase in REM sleep relative to the learners. As well, they also showed a drop in stage 2 (and by consequence number of spindles) that was not present in the learning group. The results might be interpreted as being parallel to the results in studies where the two groups did learn these tasks (Peters et al., 2008; Fogel and Smith, 2011). When the individual finds the task to be novel and has had no previous similar experience, the preferred sleep state was REM at the expense of stage 2. More difficult to understand was the significant increase in SWS. This stage has been considered to be important for declarative memory as opposed to procedural memory tasks (Gais and Born, 2004). It is possible that the adolescents in this study treated the task as declarative in part. More likely, they were showing a sleep response specific to their age group when exposed to a motor learning task. Only further studies using the same age group will clarify this.

The expected increase in spindle densities with learning, was not observed in these adolescents. This is in contrast to research which suggests that memory consolidation is linked to an increase in spindle activity (Fogel and Smith, 2011). However, as discussed, not all research points to the same conclusion. Peters et al. (2008) found that only the young adults showed this increase, whereas the older adults did not. This suggests that there are age-related differences and may be partly why our adolescents showed no significant changes in density. Peters et al. also suggested that the increase is performance dependent – only subjects who learned sufficiently, showed the expected increase in spindle activity. As discussed, our adolescents did not perform particularly well on the pursuit rotor task, and this may also help to explain the lack of relationship. Further, Fogel et al. (2007a), Schabus et al. (2008), and Fogel and Smith (2011) suggested that the changes in spindle activity are linked to intelligence, and may only be observed in individuals with higher IQ scores.

This study does contain some limitations which need to be addressed. First, we did not have a control ‘wake’ group, although we would argue that in this case one is not necessary. Our focus was on the effect of learning on sleep architecture, rather than examining how different sleep patterns affect learning. In future studies, it would be of interest to examine both effects, and in this case, having a wake group would contribute valuable information. Also, as mentioned earlier, a more captivating task may be required for this age group and may help to clarify the effect that learning procedural skills may have on later sleep. We were also limited in the sleep measures we could examine because of our take-home recording system, and future studies may want to examine sleep characteristics such as sleep efficiency and WASO, but these measures are more important for sleep disorder groups and the elderly. We did not employ sleep logs or actigraphy in our study, but the participants were a group of adolescents who were all on regimented schedules, these were not subjects who had variable sleep schedules.

## Author Contributions

Work is part of the Ph.D. thesis of RN. AM was heavily involved in the EEG scoring of the sleep data and helped in the basic design of the study. CS is the senior advisor and director of the lab, he provided basic direction for the implementation of the research study.

## Conflict of Interest Statement

The authors declare that the research was conducted in the absence of any commercial or financial relationships that could be construed as a potential conflict of interest.
